# Recent advances in understanding and managing IgG4-related disease

**DOI:** 10.12688/f1000research.9399.1

**Published:** 2017-02-23

**Authors:** Anna R. Wolfson, Daniel L. Hamilos

**Affiliations:** 1 Massachusetts General Hospital, Allergy and Immunology Division, Cox 201, 55 Fruit Street, Boston, Massachusetts, 02114, USA

**Keywords:** IgG4, plasmablasts, IgG4 related disease, IgG, Sjogren’s syndrome

## Abstract

IgG4-related disease was only recently discovered, so its description, management, and new discoveries related to its etiology are rapidly evolving. Because IgG4 itself is a unique antibody which is intimately related to the diagnosis of the disease, the role of plasmablasts in the pathophysiology remains an active area of discussion. Recent studies have uncovered a possible role for CD4-positive cytotoxic T lymphocytes, T follicular helper cells, and M2 macrophages. The clinical presentation is variable and can be vague, as this disease affects many organs and new presentations are continuing to be described. The diagnosis depends on clinical and histopathological assessment. The mainstay of treatment is with glucocorticoids, but rituximab has recently shown promise. Monitoring disease activity using imaging modalities (including positron emission tomography) and serum markers is imperative, as relapses are common. IgG4-related disease spans many medical disciplines but is a treatable condition with which all clinicians should be familiar.

## Introduction

IgG4-related disease is a recently described entity which is quite rare. Descriptions and recognition of the disease are advancing rapidly. Certainly, the fact that it closely resembles malignancy is one of the many reasons why recognizing this disease is important. Furthermore, it is generally very treatment-responsive, so early diagnosis can contribute to a significant improvement in morbidity for patients. The underlying disease mechanisms are an area of active research, and further understanding will aid in the treatment of patients.

## Epidemiology

Because IgG4-related disease was only recently described and mimics other diseases, recognition remains low and a true estimation of prevalence remains elusive. In 2011, the prevalence of autoimmune pancreatitis in Japan was 4.6 per 100,000 population ^1^, but prevalence data for other organs involved in IgG4-related disease are not well characterized. In Japanese patients with autoimmune pancreatitis as well as Japanese patients with IgG4-related disease of other organ types ^2^, the patients are predominantly male; there are 3 to 4 males to every 1 female patient. The mean age in both studies was late 60s. In a cohort from Spain with IgG4-related disease, 69.1% of patients were male and the median age at diagnosis was 53 years ^3^.

Interestingly, a recent review analyzed published pediatric cases of IgG4-related disease and found 25 cases with an average age of 13 and a mild female predominance (64%) ^4^.

In one small cohort of 25 patients with IgG4-associated cholangitis and autoimmune pancreatitis, 88% reported a history of “blue collar” work, often with exposures to occupational chemicals ^5^.

## Clinical presentation

Patients classically present with subacute, non-specific complaints, which can be general, such as weight loss, or organ-specific complaints. The classic finding is a tumefactive lesion in at least one organ, but the disease can develop over years, adding new organs one at a time, so patients may present with multi-organ disease ^6^. In the study by Chen *et al*., the mean number of organs involved was 3.0 ± 1.6 (range of 1–10) ^7^. If the disease has progressed, organ failure can occur. Ruling out malignancy is crucial when the diagnosis of IgG4-related disease is considered. The differential is broad and is largely organ-specific, but multi-centric Castleman’s disease and other multi-organ autoimmune diseases such as granulomatosis with polyangiitis, Sjögren’s syndrome, eosinophilic granulomatosis with polyangiitis, and sarcoidosis should be considered.

**Figure 1.**
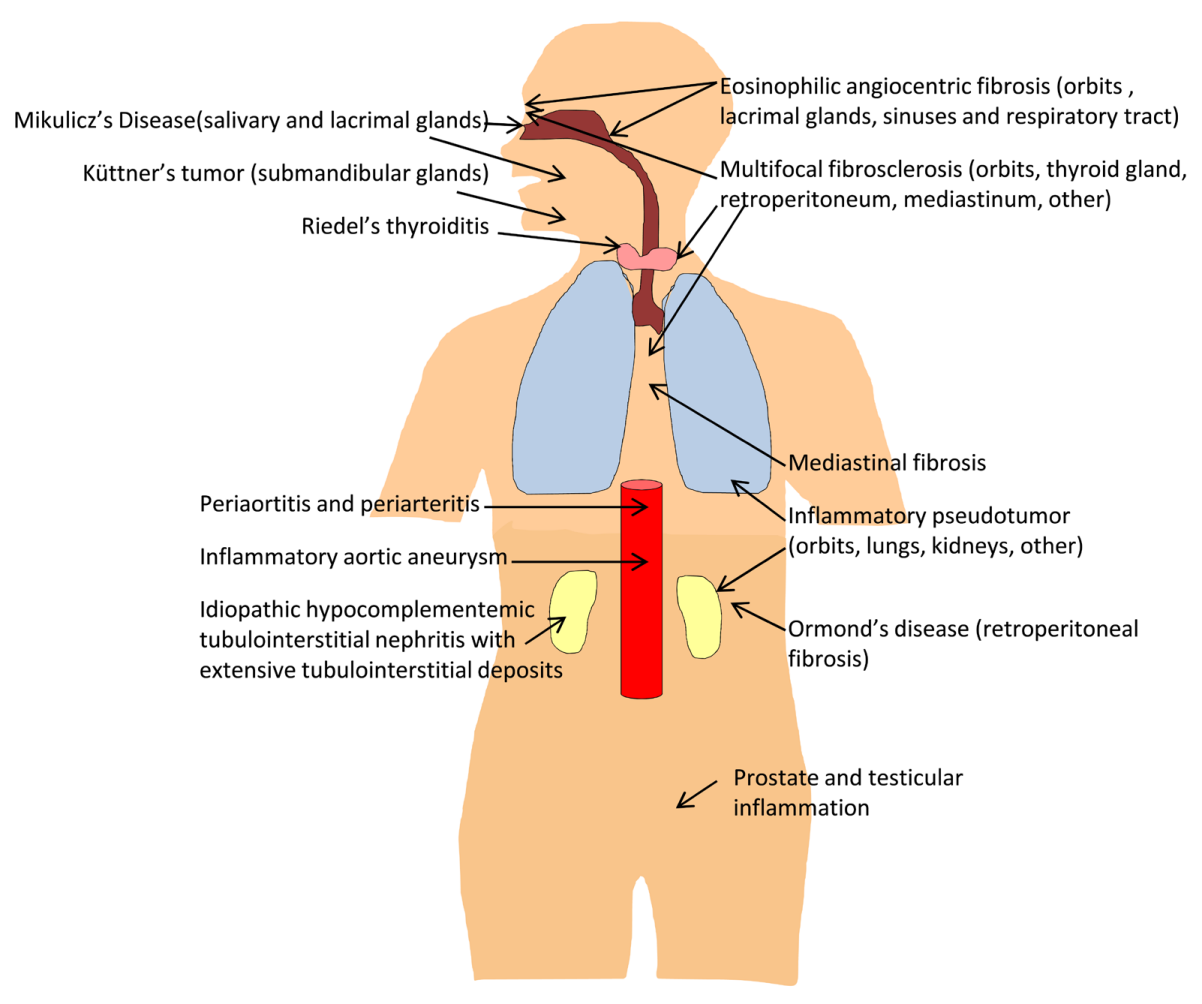
Many organs are involved in IgG4-related disease. This figure shows diseases that have now been re-classified to be part of IgG4-related disease, including eponymous names.

Although the classic organ involved in IgG4-related disease is the pancreas, which presents classically with obstructive jaundice due to damage to the biliary tree, many organs can be involved ( Figure 1). Recent novel reports of IgG4-related disease include disease involving the lung presenting with cough, dyspnea, chest pain, and fever ^8^; involving the sinuses, which in some cases can be invasive ^9– 11^; involving the esophagus presenting with dysphagia ^12^; involving the prostate presenting with urinary retention ^13^; or involving the testes presenting with scrotal pain ^14^. Skin manifestations were rare in the series reported by Chen *et al*. ^7^. A review of the dermatological literature showed that the most common skin presentations are papules, plaques, and nodules of the head and neck ^15^. Clinical symptoms of patients were well summarized by Chen *et al*. ( Table 1) ^7^. Organ involvement from three case series is summarized in Table 2.

**Table 1.** Clinical manifestations of IgG4-related disease, as summarized in Chen *et al*. (out of 200 cases, 91% Chinese) ^7^.

Clinical sign or symptom	Percentage reporting sign or symptom in Chen *et al*. ^7^
Submandibular gland swelling	51.0%
Lacrimal gland swelling	42.0%
Superficial lymph node enlargement	37.0%
Abdominal pain	35.0%
Parotid gland swelling	24.0%
Nasal congestion	21.5%
Jaundice	14.0%
Pruritus	13.5%
Cough	13.5%
Lower back pain	13.5%
Dysuresia	13.0%
Dry mouth/dry eye	12.5%
Nausea or vomiting or both	12.0%
Fever	9.0%
Edema	8.5%
Exophthalmos	5.5%
Arthralgia	4.0%
Thyroid enlargement	2.5%
History of allergy	59.0%
Asymptomatic	1.0%

**Table 2.** Comparison of organ involvement in three large case series.

Organ	Chen *et al*. ^7^, percentage of 200 cases ^a^	Fernandez- Codina *et al*. ^3^, percentage of 55 cases ^b^	Inoue *et al*. ^2^, percentage of 235 cases ^c^
Lymph nodes	56.5%		
Adenopathic		2%	
Salivary glands		16%	34%
Submandibular gland	51.0%		
Parotid gland	24.0%		
Pancreas	38.5%	16%	60% ^c^
Lung	32.0% ^d^	9%	13%
Pleura		4%	
Sinus	21.5%		
Maxillary		15%	
Bile ducts/biliary	19.0%	4%	13%
Retroperitoneal tissue	18.0%	27%	4%
Prostate	13.0%	0%	
Kidney	10.0%	7%	23%
Artery	7.0%	0%	4%
Gallbladder	5.5%	5%	
Skin	5.5%	0%	
Inflammatory pseudotumor	5.0%		
Liver	3.0%	0%	
Orbit			4%
Orbitary pseudotumor		22%	
Extraocular muscles		2%	
Lacrimal gland	42.0%	15%	23%
Aorta		9%	20%

Chen *et al*. had predominantly Chinese cases ^7^, Fernandez-Codina *et al*. ^3^ analyzed patients from a Spanish registry ^6^, and Inoue *et al*. studied a Japanese cohort ^2^. ^a^Chen *et al*. included those who fulfilled 2011 diagnostic criteria for definite, probable, or possible IgG4-related disease ^7^. ^b^Fernandez-Codina *et al*. used 2012 international diagnostic consensus on IgG4-related disease for their inclusion ^6^. Fernandez-Codina *et al*. also reported 2% involvement of the thyroid, breast, pericardium, or mediastinum; 7% involvement of the mesentery; 4% involvement of the pachymeninges; and 0% involvement of the hypophysis. ^c^Inoue *et al*. included patients with a diagnosis of type I autoimmune pancreatitis as well as those with IgG4-related disease on pathology, which is possibly what accounts for a greater number of patients with pancreatic involvement ^2^. Inoue *et al*. reported 5% involvement of the paravertebra and 3% other sites. ^d^In Chen *et al*., this includes parenchyma, airway, and pleura.

Although most patients present before multi-organ involvement is evident, Higashioka *et al*. reported a case of a patient who had 10 organs involved in IgG4-related disease at the time of his diagnosis ^16^.

The clinical presentation of IgG4-related disease often mimics a tumor, which can result in unnecessary surgical intervention. Surgical resection for presumed malignancies occurred in 18 out of 53 patients, about 34%, who were ultimately diagnosed with IgG4-associated cholangitis in the series reported by Ghazale *et al*. ^17^. For example, Park and Kim ^18^ and Wang *et al*. ^19^ reported a case in which the discovery of a mass in the renal parenchyma resulted in a patient undergoing nephrectomy, and ultimately the mass was found to be IgG4-related disease. Similarly, Kim *et al*. presented a case of a patient in whom there was concern for cholangiocarcinoma and who underwent a left trisectionectomy but was ultimately diagnosed with IgG4-related disease ^20^. IgG4-related disease can also be initially mistaken for lymphoma, as both diseases can present with lymph node swelling as an initial symptom ^21, 22^. Differentiating IgG4-related disease from cancer is of the utmost importance. The diagnosis of IgG4-related disease is ultimately reliant on diagnostic tools ( Table 3).

**Table 3.** Evolution of diagnostic criteria for diagnosis of IgG4-related disease.

**Autoimmune pancreatitis diagnosis** **using the HISORt ^a^ criteria, 2007 ^73^**	Obstructive jaundice, pancreatic mass/enlargement, or pancreatitis with at least one of the HISORt criteria:	
	Histology	Lymphoplasmacytic infiltrate with storiform fibrosis or immunostaining shows abundant IgG4-positive cells.
	Imaging	Diffusely enlarged pancreas and a diffusely irregular, narrow pancreatic duct
	Serology	Elevated IgG4 levels
	Other organ involvement	Extrapancreatic manifestations
	Response to therapy	Manifestations resolve with steroid therapy
**IgG4-associated cholangitis diagnosis** **using the HISORt criteria, 2008 ^17^**
	Histology of bile duct	Lymphoplasmacytic sclerosing cholangitis on resection specimens
	Imaging	Strictures of the bile ducts
	Serology	Elevated IgG4 levels
	Other organ involvement	Pancreas, kidney, retroperitoneum, salivary glands
	Response to therapy	Normalization of liver enzyme levels or resolution of stricture
**Comprehensive diagnostic criteria for** **IgG4-related disease, 2011 ^74^**
	Definite: 1+2+3. Probable: 1+3. Possible: 1+2.	
	1. Clinical examination showing characteristic diffuse/localized swelling or masses in single or multiple organs	
	2. Hematological examination shows elevated serum IgG4 concentrations (135 mg/dL)	
	3. Histopathologic examination shows (1) Marked lymphocyte and plasmacyte infiltration and fibrosis (2) Infiltration of IgG4 ^+^ plasma cells: ratio of IgG4 ^+^ to IgG ^+^ cells >40% and >10 IgG4 ^+^ plasma cells per high-power field	

In 2007, Chari published criteria to diagnose autoimmune pacreatitis ^73^, and Ghazale *et al*. followed in 2008 with criteria to diagnose IgG4-associated cholangitis ^17^; both were the Mayo Clinic experiences. Ultimately, in 2011, comprehensive criteria for diagnosing IgG4-related disease were developed by Umehara *et al*., from Japan ^74^. ^a^HISORt, histology, imaging, serology, other organ involvement, and response to therapy.

## Monitoring and following disease

### Serum studies

Although IgG4 levels are classically elevated in IgG4-related disease (Japanese criterion is more than 135 mg/dL), this finding is not necessary to make the diagnosis—which is based on histopathological findings when histopathology is available, as obtaining specimens (for example, in the pancreas and biliary tract) is not without risk—and is not specific to the disease ^23^. Indeed, approximately 30% of patients with IgG4-related disease have normal IgG4 serum levels ^23^ (the upper limit of normal for serum IgG4 is 135 mg/dL ^6^), but the greater the number of organs involved in the disease, the higher the elevation in the serum IgG4 level ^24^. An elevated IgG4 level is also not specific for the disease, as there are many diseases associated with this laboratory abnormality; it similarly does not have a good positive predictive value ^25^. Importantly, Oseini *et al*. showed that elevated IgG4 can be seen in patients with cholangiocarcinoma ^24^, and Ghazale *et al*. showed that elevated IgG4 cannot be used to distinguish autoimmune pancreatitis from pancreatic cancer, thus elevated serum IgG4 levels do not exclude these deadly differential diagnoses ^26^. Patients with primary sclerosing cholangitis can also have elevated IgG4 levels ^27, 28^. However, 65 out of 72 patients with IgG4-related disease had elevated IgG4 levels, yielding a sensitivity of 90% according to a study by Carruthers *et al*. ^25^.

With glucocorticoid treatment, elevated IgG4 levels can decrease but may remain above the upper limit of normal ^23^. In one study of the treatment of autoimmune pancreatitis, the IgG4 level remained above the upper limit of normal in 115 out of 182 patients (63%) treated with glucocorticoids ^29^. Furthermore, despite elevated levels, patients may remain in remission; in this study, only 30% of patients with persistent elevation of serum IgG4 had a relapse ^29^. Although an increase in IgG4 level may be a marker of relapse in some patients, for 10% of the patients in this study, relapses also occurred without an increase in the serum concentration of IgG4 ^29^.

Although it is not yet clinically available, quantitation of circulating plasmablasts using flow cytometry is more sensitive than serum IgG4 levels, so this measurement could emerge as a better test for the diagnosis and monitoring of IgG4-related disease in the future ^30, 31^.

IgG4/IgG RNA ratio is a novel, highly accurate marker for IgG4-related disease and has been validated for IgG4-associated cholangitis and autoimmune pancreatitis ^32^. The clinician should be aware that very high serum IgG4 levels may lead to errors in the measurement of IgG4 serum concentrations, resulting in reporting of spuriously low values, a type of “prozone” effect ^33^.

In conclusion, IgG4 levels alone are insufficient to make a diagnosis of IgG4-related disease or to monitor patients in follow-up. Elevations in eosinophils and C-reactive protein can also be observed in patients with IgG4-related disease, but these are non-specific ^6^.

### Imaging

As already stated, histopathology is an integral part of the diagnosis, but imaging can aid in defining the disease involvement and in follow-up. A recent study showed the efficacy of using positron emission tomography/computed tomography (PET/CT) with the glucose analogue 2-[(18)F]-fluoro-2-deoxy-d-glucose (FDG) in patients diagnosed with one-organ involvement of IgG4-related disease to assess for the involvement of other organs ^34^. In this study, Zhang *et al*. reported that 9 out of 10 patients initially enrolled in the study with single-organ disease were ultimately found to have multi-organ disease ^34^. PET/CT is also useful for monitoring disease progression and identifying sites for biopsy ^35^. CT and magnetic resonance imaging (MRI) can also be used to identify organ enlargement, and lesions identified by CT typically generate low signal intensity in T2-weighted MRI ^36, 37^.

## Histopathology of IgG4-related disease

Histopathology is the gold standard for diagnosis of IgG4-related disease, since the disease cannot be diagnosed on the basis of IgG4 levels in the serum or in tissue samples on pathology, although these findings are suggestive of the disease. The classic findings are dense lymphoplasmacytic infiltrate, storiform fibrosis, and obliterative phlebitis (which means partial or complete obliteration of a vein) ^38^. ****The presence of at least two of these findings in combination with infiltration of IgG4-positive plasma cells is highly suggestive of a diagnosis of IgG4-related disease, with the cutoff value for the number of IgG4-positive plasma cells being defined for each organ. Eosinophils are commonly present. IgG4-positive plasma cells are distributed diffusely throughout the tissue, even in patients who have normal levels of serum IgG4. This finding can help to differentiate IgG4-related disease from other diseases characterized by plasma cell infiltration but is not sufficient to make the diagnosis. The cutoff value for the number of IgG4-positive plasma cells required to make a diagnosis is specific to the organ, but the ratio of IgG4 to IgG cells must be greater than 40% ^38^.

It is worth mentioning that the clinician must be cautious to not over-emphasize the role of histopathology, especially IgG4 immunostaining, when making a diagnosis of IgG4-related disease. Strehl *et al*. highlighted this nicely: they analyzed 121 randomly selected pathology specimens with lymphoplasmacytic infiltrates ^39^. Interestingly, variably high numbers of IgG4-positive plasma cells were found in diverse samples, particularly in rheumatoid synovitis, carcinoma-associated inflammation, and sclerosing sialadenitis ^39^. This article thus highlights the fact that IgG4 can be elevated in inflammation unrelated to IgG4-related disease ^39^. Cheuk and Chan also warned of the danger of over-interpretation of lymphadenopathy with positive immunostaining for IgG4 in a patient with no other clinical or laboratory signs of IgG4-related disease ^40^.

## Treatment options

### Watchful waiting

Although organ involvement in IgG4-related disease requires treatment, less aggressive management is appropriate if this is not the case. For example, in asymptomatic lymphadenopathy, watchful waiting is acceptable.

### Glucocorticoids

The first-line treatment of IgG4-related disease is glucocorticoids ^29^, and the first prospective, randomized, placebo-controlled trial examining their efficacy for the treatment of autoimmune pancreatitis was just published by Masamune *et al*. ^41^. In this study, remission was induced with prednisolone in both groups and then withdrawn at 26 weeks in 19 of the patients versus continued for 3 years in 30 patients ^41^. The authors found that the relapse rate was significantly lower in the maintenance group, where 7 out of 30 patients (23.3%) relapsed compared with the cessation group, where 11 out of 19 (57.9%) relapsed ( *P* = 0.011) ^41^.

The initial treatment for autoimmune pancreatitis is typically prednisolone 0.6 mg/kg for 2 to 4 weeks, which is then tapered every 2 to 4 weeks to 5 mg/day over the subsequent 3 to 6 months, and then 2.5 to 5 mg/day is continued for 3 years ^42, 43^. Notably, low doses of prednisone may be as effective as higher doses for induction therapy: a retrospective review found that outcomes were not significantly different in patients who received prednisone in the range of 10 to 60 mg per day ^44^. Clinical symptoms, imaging, and blood tests are used to guide the taper ^42^. Many patients have an effective, rapid response to glucocorticoids, and an alternative diagnosis should be considered if this rapid response is not seen ^42, 45^. Advanced fibrosis, however, is a poor prognostic sign ^42^.

A recent systematic review by Brito-Zeron *et al*. reflects the common paradigm with treatment with glucocorticoids: 1,186 out of 1,220 patients (97%) who received monotherapy with glucocorticoids as their first-line drug had a therapeutic response, but the response was classified as complete in only 84 out of 130 patients (65%) in whom this information could be extracted ^45^. This study also discussed the management of relapses, where glucocorticoids were most commonly used but immunosuppressive agents were used in 149 cases (39%) (azathioprine in 126 out of these 149 cases) and rituximab in 9 cases (2%) ^45^. The treatment was reported to be effective to treat the relapses for 219 out of 231 cases (95%) treated with glucocorticoids, 56 out of 69 patients (81%) treated with azathioprine, 16 out of 22 patients (72%) treated with other immunosuppressives, and 9 out of 9 patients (100%) treated with rituximab ^45^.

### Alternatives to glucocorticoids

Steroid-sparing treatments, such as azathioprine, mycophenolate mofetil, and methotrexate, are used to allow respite from the side effects of glucocorticoids and in order to maintain remission, but evidence for their effectiveness is lacking ^42^. A more recent, promising option is treatment with rituximab; the mechanism is via the depletion of CD20-positive plasmablast precursors— with fewer plasmablasts, IgG4 production decreases ^46^. Yamamoto *et al*. recently published a case report of successful treatment with abatacept (an inhibitor of T-cell activation), but treatment with this is in its infancy ^47^.

### Alternatives to medication

Surgery and radiotherapy have been reported to treat tumefactive masses in the pancreas, kidneys, or other organs in cases where the diagnosis of IgG4-RD may not have occurred until after histopathologic analysis was performed. However, these modalities have had some role in treating patients with specific involvements, such as infiltrative masses of tubular structures, such as the biliary tract, lymphadenopathy, or masses in other solid organs. In the recent systematic review of treatment of IgG4-related disease, out of 1,952 patients, 1,437 (74%) were treated with glucocorticoids as first-line, but 213 patients (11%) were treated with surgery or radiotherapy and 38 (2%) were treated with other options ^45^. Surgery was considered effective in 14 out of 17 patients (82%), and radiation was considered effective in 9 out of 12 patients (75%) ^45^. Combination surgery and glucocorticoids were effective in 20 out of 22 patients (91%) ^45^.

## Mortality

In a recent systematic review of treatment of IgG4-related disease, Brito-Zeron *et al*. compiled the mortality data from 7 studies for 294 patients and a mean follow-up of 29.2 months ^45^. In this group, mortality was reported in 26 patients (8.8%). Four patients died of pulmonary disease, 1 died of an aneurysm, 1 of cholangitis, 1 of renal failure, 7 of cancer, 4 of cardiovascular disease, 3 of infection, and 5 of unknown or other causes ^45^.

## Pathophysiology of IgG4

### IgG4 molecule

The immunoglobulin IgG4 isotype accounts for less than 5% of the total IgG in healthy patients and is the least abundant of the IgG subclasses ^48^. It is unique among the immunoglobulin isotypes in that it undergoes a process known as fragment antigen-binding (Fab)-arm exchange. The disulfide bonds between the heavy chains of the IgG4 molecules are unstable, so dissociation of the heavy chains occurs, leading to random recombination. The result is antibodies with two different antigen-binding specificities that are unable to cross-link antigen or form immune complexes in a manner similar to that of IgG1, IgG2, and IgG3 antibodies. IgG4 antibodies are also unable to fix complement ^42, 48, 49^. IgG4 has traditionally been regarded as anti-inflammatory.

Another unique property of IgG4 is that it sometimes binds the Fc region of IgG, producing a rheumatoid factor (RF), but the physiologic relevance of this is unclear ^50^. RF was reported to be positive in 20% of patients (50 out of 255) with IgG4-related disease ^6^. The question of whether IgG4 itself is pathogenic remains to be fully elucidated. Shiokawa *et al*. injected Balb/c mice with IgGs from patients with IgG4-related disease in order to explore this question ^51^. Pancreatic injury was seen with both IgG1 and IgG4, but more so from IgG1. Interestingly, patient IgG1-induced damage to the mouse pancreas was somewhat attenuated with the addition of patient IgG4.

## Genetics

There is much still to be learned about the genes involved in IgG4-related disease. Genetic susceptibility has been associated with certain human leukocyte antigen (HLA) haplotypes in Japanese and Korean populations ^49^. More recent studies have identified genes involved in pancreatic cell injury ^52^, and cytotoxic T-lymphocyte antigen 4 (CTLA-4) gene polymorphisms have been associated with IgG4-related disease in the pancreas ^53^.

## Autoimmunity

Autoimmunity has long been hypothesized as a pathologic mechanism in IgG4-related disease, but studies have failed to identify a clear autoantigen specific to the disease that selectively induces the production of IgG4 autoantibodies ^49^. It stands to reason that the target of the antibody would be specific to the organs commonly affected in IgG4-related disease. Indeed, a recent study which sought to identify autoantigens in IgG4-related disease identified only one candidate: prohibitin ^54^. As prohibitin is involved in anti-proliferation, the authors hypothesize that antibodies against prohibitin would result in tissue growth and the presentation of IgG4-related disease ^54^. Another recent study showed that anti-nuclear antibody (ANA) in IgG4-related disease was not of the IgG4 subclass ^55^. ANA was reported to be positive in 32% of patients (168 out of 524 patients) ^6^.

## Innate immunity

Evidence is beginning to accumulate that there is a role for innate immunity in the mechanism of IgG4-related disease. Nakajima *et al*. used DNA microarray analysis to show that the expression of genes associated with allergy or innate immunity is lower in the peripheral blood mononuclear cells of patients with IgG4-related disease compared with healthy controls (HCs) and that these levels were increased with steroid therapy ^56^. Furthermore, Watanabe *et al*. recently postulated that since activation of the innate immune system leads to the production of IgG4, this pathway could be the target of future treatments for IgG4-related disease ^57^.

## Hypocomplementemia

A feature that is important in IgG4-related disease is hypocomplementemia. The etiology of this was studied by Sugimoto *et al*., who showed that patients with hypocomplementemia in IgG4-related disease have a high serum level of C1q-binding IgG4, thus implying that IgG4 participates in the activation of complement in these patients by an unknown mechanism ^58^.

## Role of T cells (Th2, Treg, Tfh, and CD4 ^+^ CTL)

It has been observed that CD4 T cells are present at the sites of inflammation in IgG4-related disease. Similar to IgE, class switching to IgG4 depends on interleukin-4 (IL-4) and IL-13. These interleukins are considered part of the type 2 helper T (Th2) immune response. Messenger RNA levels of these Th2 cytokines are higher in organs affected by IgG4-related disease in comparison with other classic autoimmune conditions ^59, 60^. The peripheral blood lymphocytes of patients with IgG4-related disease also express elevated levels of Th2 cytokines ^60^. A subset of patients also has elevated eosinophils and IgE, suggestive of an underlying disease mediated by Th2 cytokines.

Allergens and nematodes that induce IgE also induce IgG4. However, a so-called “modified Th2 response” refers to the scenario in which IgG4 antibody is produced without IgE antibody production, which seems to be the desired, healthy response to innocuous antigens, as it occurs during occupational exposures to antigens such as in beekeepers ^48^. Allergen-specific IgG4 antibodies are also induced during allergen immunotherapy and accompany clinical improvement ^61^.

A role for elevated levels of regulatory T (Treg) cells in IgG4-related disease has also been suggested ^62^. This is in contrast to most autoimmune conditions, in which a deficiency in Treg cells is present and thought to promote autoimmunity. In IgG4-related disease, it has been hypothesized that collagen deposition occurs through the production of cytokines produced by Th2 and Treg cells and thus that these cells could act synergistically to induce tissue fibrosis, a prominent feature of the disease ^63^.

Recent studies ^64, 65^ suggest that the role of Th2 immune responses in IgG4-related disease may be overstated and may, in fact, be a reflection of atopy, which affects approximately 40% ^66^ of patients with IgG4-related disease, which is similar to the prevalence in the general US population. Instead, evidence is accumulating for a role of CD4-positive cytotoxic T lymphocytes (CTLs). Maehara *et al*. have shown over-expression of genes associated with CD4-positive CTLs in the salivary glands of patients with IgG4-related disease compared with HCs and compared with patients with Sjögren’s syndrome and chronic sialoadenitis ^65^. Furthermore, CD4-positive CTLs, which produce IL-1β, transforming growth factor-beta 1 (TGF-β1), and interferon-gamma (IFN-γ), are found in affected tissue. IL-1β and TGF-β1 have been shown to be involved in the pathology of fibrosis ^67^, an integral feature of IgG4-related disease, thus further strengthening the putative role of CD4-positive CTLs. Finally, rituximab-mediated B-cell depletion results in both clinical improvement of patients’ IgG4-related disease and a decrease in the number of CD4-positive CTLs ^64^. This new research is exciting, as CD4-positive CTLs may ultimately prove to be the major mechanism of pathogenesis of IgG4-related disease.

The role of circulating follicular helper T (Tfh) cells in IgG4-related disease is an active area of research. Akiyama *et al*. have published two articles implicating a role for these cells in IgG4-related disease ^68, 69^. They showed that in patients with untreated IgG4-RD compared with those with primary Sjögren’s syndrome, those with multi-centric Castleman’s disease, and HCs, the number of circulating Tfh2 cells (defined as CD3 ^+^CD4 ^+^CD45RA ^−^CXCR5 ^+^CXCR3 ^−^CCR6 ^−^) was significantly increased. Furthermore, “activated” Tfh2 cells (defined by the additional markers CCR7 ^low^PD-1 ^high^) were shown to induce IgG4 production and to decrease in response to treatment with glucocorticoids in parallel with decreased disease activity ^68^. Activated circulating Tfh1 cells were also found to correlate with disease activity but not with serum IgG4 levels ^68^.

## Pathogenic effects of B cells and IgG4

Maillette de Buy Wenniger *et al*. showed that IgG4-positive B cells were clonally expanded in patients with IgG4-associated cholangitis and decreased when the patients were treated with glucocorticoids ^70^. The role of the B cells in IgG4-related disease is especially interesting given the utility of rituximab-mediated CD20 ^+^ B-cell depletion in treating these patients ^46^. Recent studies have shown that circulating plasmablasts are increased in patients with IgG4-related disease and that their levels reflect disease activity ^30, 31^. The plasmablasts are oligoclonal, which suggests that specific antigen(s) may be driving their production ^30^. In B-cell development, plasmablast differentiation occurs between activated B cells and plasma cells.

## Role of M2 macrophages and MARCO

Returning to the issue of the IgG4-related disease being mainly a Th2-mediated disease, a possible disease mechanism could be mediated by M2 macrophages, which are defined as alternatively activated macrophages stimulated by Th2 response. IL-10, IL-13, and CCL18 produced by M2 macrophages induce fibrosis, and a recent study ^71^ showed that there are increased numbers of M2 macrophages in submandibular glands affected by IgG4-related dacryoadenitis and sialoadenitis and that fibrosis is associated with an increased number of M2 macrophages. Macrophage receptor with collagenous structure (MARCO) is a pattern-recognition receptor with an expression pattern similar to that of the M2 macrophage marker CD163. It has been speculated that MARCO may be involved in the initiation of IgG4-related disease ^72^. Future research is needed to ascertain whether there is any interaction between CD4-positive CTL or Th2 Tfh cells and M2 macrophages in promoting IgG4-related disease.

## Conclusions

IgG4-related disease, though uncommon, is an important disease entity to consider in the differential of a patient presenting with tumefactive lesions. Current research into the underlying pathophysiology is focused on improved understanding of the T-cell and B-cell compartments. Future studies to improve the prognosis of this condition will focus not only on arresting the abnormal immunologic response but also on treating fibrosis.
